# Risk buffering and resource access shape valuation of out-group strangers

**DOI:** 10.1038/srep30435

**Published:** 2016-07-29

**Authors:** Anne C. Pisor, Michael Gurven

**Affiliations:** 1 Department of Anthropology, University of California, Santa Barbara, CA 93106-3210, USA.; 2 Department of Human Behavior, Evolution, and Culture, Max Planck Institute for Evolutionary Anthropology, Deutscher Platz 6, D-04103 Leipzig, Germany.

## Abstract

Unlike other primates, humans exhibit extensive inter-group tolerance and frequently build relationships with out-group members. Despite its common occurrence, little is known about the conditions leading to out-group relationship building in humans. What are the social and ecological factors promoting valuation of out-group members as potential social partners? Do they differ from those promoting valuation of in-group members? We propose that opportunities for non-local resource access and resource buffering, crucial in the human foraging niche, will increase valuation of out-group strangers. Using survey and experimental data collected among three Bolivian horticultural populations, we find that individuals with fewer non-locally available resources and more information about out-groups demonstrate more generosity toward out-group strangers, but not in-group strangers. The effects are specific to subjective resource access, not objective measures of access, and out-group exposure, not stereotypes. Further, depending on the measure, existing network connections affect both out-group and in-group giving, suggesting that new partnerships from both in-groups and out-groups may bolster one’s networks. Our results illustrate how evolved human psychology is sensitive to the costs and benefits of both out-group and in-group relationships, but underscore that the social and ecological factors favoring new relationships with in-group versus out-group strangers may differ.

Humans have a long history of interaction with individuals from different places and cultural backgrounds. While much research on inter-group relationships in human evolution has focused on competition and conflict^1^,^2^,^3^,^4^, out-groups as sources of danger^5^,^6^, and in-group favoritism at the expense of out-groups^7^, evolutionary researchers have paid little attention to the conditions that favor building connections with individuals from different ethnic groups, individuals who practice different modes of production, or – more recently in human history – individuals from different religious groups (cf.^8^,^9^,^10^,^11^,^12^). Chimpanzees and other primates are mostly indifferent or hostile towards strangers from other groups^13^, yet archaeological and ethnographic evidence provide many examples of relationship building with out-groups throughout human history, as facilitated by marriage, trade, and friendship^14^,^15^,^16^,^17^,^18^,^19^,^20^. Between-group relationships served functional roles, including improvement of non-local resource access, resource buffering, and information transmission. Among contemporary industrialized populations, social psychologists and behavioral economists have likewise documented higher valuation for out-group members – measured as cooperation with, trust in, or empathy toward out-group individuals – in contexts where competition is low and mutually beneficial interactions are possible^8^,^21^,^22^,^23^. Furthermore, an actor is more likely to value out-group members when her in-group is of low status^24^,^25^, a correlate of poor resource acquisition in situations of resource scarcity^26^.

Taken together, these findings suggest that positive valuation of out-group members as potential social partners is governed by an evolved human psychology sensitive to the expected benefits and costs of interaction with others, not unlike the system that governs partner choice and alliance formation with in-group members^12^,^27^. As with in-group valuation, the expected benefits and costs of interaction with out-groups may be informed by a participant’s own observations, past interactions with out-groups, and socially transmitted information^28^,^29^. When the expected net benefits of interaction are sufficiently high, an actor should be more willing to display tolerance of and cooperative intent towards out-group members, thereby investing in her reputation as a potential social partner^30^,^31^,^32^,^33^. Higher valuation of out-group individuals may improve inter-group relations in turn, increasing the likelihood of large-scale collaboration^34^ and reducing parochial behavior^35^,^36^.

When might out-group relationships have expected net benefits for an actor? Humans evolved as hunter-gatherers, dependent on a foraging ecology with return rates that varied across space and time. In-group relationships provide crucial buffers to shortfalls caused by illness and food production failure in isolated small-scale populations^33^,^37^. These relationships remain important in populations integrating to national markets^38^,^39^ and industrialized populations^40^, as reliance on credit is not a perfect buffer^41^ and resource shortfalls are associated with lower fertility^42^,^43^. Likewise, out-group relationships may buffer shortfalls due to production failures, illness, and other idiosyncratic shocks, but they also expand on the buffering possible with in-group networks, as they can provide non-local resource access and buffer aggregate shocks impacting all members of an actor’s local network^14^,^15^,^17^,^19^. For example, when local sources of water would run out in the 1970s, the San would disperse, calling upon social connections up to 150 or 200 km away^44^. Historically and prehistorically the San also relied on members of different ethnic groups for resource access, trading locally produced goods for pottery, hunting dogs, and iron tools^16^.

In the present paper, we suggest that while need and insufficient buffering in existing networks should motivate investment in new relationships, whether with in-group or out-group strangers, the need or desire for non-local resource access should affect valuation for new out-group relationships more than for new in-group relationships. Further, expectations of the gains to be reaped via out-group relationships may be altered by past observations of, experience with, or socially transmitted information about out-groups. We investigate out-group and in-group valuation in relation to non-local resource access among three populations of horticulturalists from lowland Bolivia. Further, we explore the relative roles of social support, as well as past experience with out-groups and stereotypes about their resource access, in modulating out-group and in-group valuation.

We predict that:

(P1) Lack of access to non-local resources will increase valuation for out-group members. In lowland Bolivia, this includes lack of access to monetary income and market items, as well as a participant’s *subjective* socioeconomic status, or her perceived lack of access to money and market items.

(P2) Existing information about out-group members, particularly positive information related to the potential for cooperative outcomes, will increase her valuation for them. Relevant sources of information include socially transmitted information, such as stereotypes, and an actor’s own past exposure, such as number of places visited, number of places lived, or hours spent watching TV or movies.

(P3) An actor’s current state of need and the degree to which she can rely on her existing social networks will not differentially affect her out-group valuation in relation to her in-group valuation. In the lowland Bolivian context, her degree of need includes her food insecurity, whether her recent produce and monetary income were below normal, household dependency, and illness in the last month. Her network support is proxied by being able to borrow from communities where she has lived previously, or stay with a member of these communities during a hypothetical flood.

As a proxy for out-group and in-group valuation, participants played a non-anonymous economic game in which they could be generous toward strangers from the participant’s self-identified in-group or out-group; recipients learned the donor’s name and amount sent. The lack of anonymity in the game was designed to mimic the opportunity to make an initial investment in a new relationship with a stranger.

The data analyzed here were collected among the Mosetén, the Tsimane’, and a multicultural community nicknamed “Intercultural.” Members of these three populations have differential exposure to out-group members and markets, which may boost the benefits to be gained from out-group relationships. These differences in exposure are not solely between-group differences, but include substantial within-group differences in market integration and out-group contact; as such, we studied the three populations together to take advantage of a broader range of variability, but in the context of a roughly similar socioecological, cultural, and political environment (see Supplementary Methods 1).

## Results

**Figure 1:**
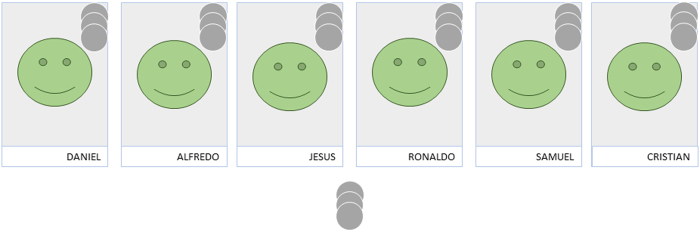
An example of the non-anonymous giving game (NAGG). Six photos were arrayed on a table, all of past participants in the experiment. The three men on the left were participants who identify with one ethnic group, for example, and the three on the right with another. Participants were told the name and group affiliation of each individual. Stacks of three one boliviano coins were placed on each photo and in front of the participant. The participant could move any number of coins between photos, from photos to his own stack, or from his stack to the photos. Participants were informed that any coins left on a photo would be given to that person in the participant’s name (unless the participant wished to remain anonymous) and any coins left in front of the participant would be his.

Each participant was presented with three photos of in-group and three photos of out-group strangers, and was given 21 *bolivianos* (1/3 of a day’s wage; $0.14/B1) to allocate among these six individuals and herself (Fig. 1). Participants gave away an average of 74% (B15.53) to the six candidate recipients, keeping the remainder for themselves (B5.47; SD = 5.77). They sent an average of 14% of the stakes (B2.94; SD = 1.56) to each in-group stranger, more than the 10% average stakes (B2.20; SD = 1.50) sent to each out-group stranger (t = 4.27, p < 0.001). Though we designed the economic game such that recipients would learn the name of donors, 33% of our sample opted not to share their names with recipients. Other descriptive statistics appear in Supplementary Table 1. Table 1 summarizes our predictions, the proxies used for testing each prediction, and model results.

### (P1) Existing non-local resource access

**Figure 2:**
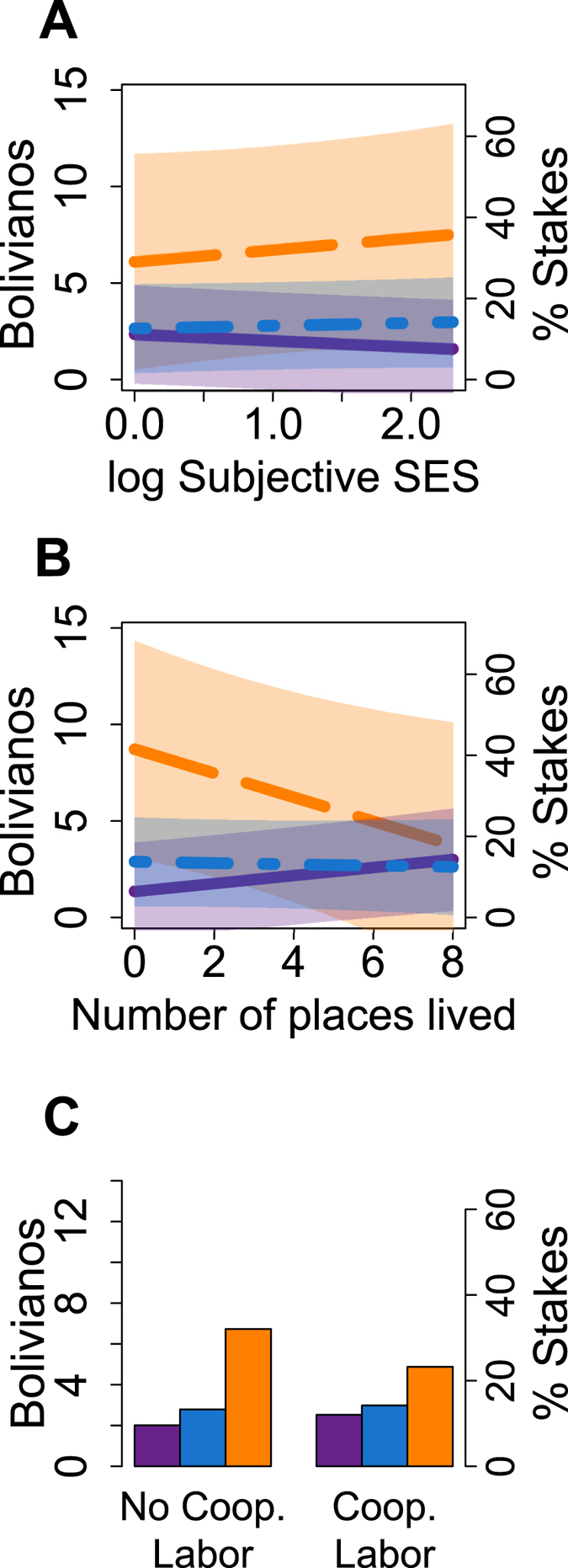
Predicted amounts allocated (with 95% prediction intervals for **a** and **b**) to an out-group member (purple, solid line), an in-group member (blue, short-long dash), and the self (orange, long dash) by (**A**) log subjective socioeconomic status, (**B**) number of places lived, and (**C**) availability of cooperative labor partners.

For proxies of non-local resource access, higher values mean *pre-existing* access to non-local resources. Participants who had higher subjective socioeconomic statuses relative to others in their community (i.e., believed themselves to have more money and market-purchased items relative to others in their communities) were gave less money to out-group strangers (−B0.33 for each one unit increase on the log scale, p < 0.10; Table 2) but did not differ in money sent to in-group strangers (B0.14, p = 0.52) or money kept for themselves (B0.60, p = 0.44; Fig. 2A; Supplementary Table 3). Household income had no significant effect on giving. The dollar value of market items owned had no significant effect across populations, but within the Tsimane’ subsample, individuals who had more market item assets gave significantly less to out-group strangers than Tsimane’ participants who had fewer market assets (−B2.72 per standard deviation increase in market items, p < 0.01; Supplementary Table 4).

### (P2) Past exposure

Participants who had lived in more locations gave significantly more money to out-group strangers relative to those who had lived in fewer locations (B0.21 for each additional location, p < 0.01), keeping less for themselves (−B0.62, p < 0.10; Fig. 2B). Those who had watched more TV or movies in the past week also gave more to out-group strangers relative to those who had watched less (B0.23 for each standard deviation of watching, p = 0.10), keeping less for themselves (−B1.06, p < 0.10). There was no effect of number of cities and towns visited over one’s lifetime on giving.

### (P2) Stereotypes

Due to small sample size, separate models were fit to investigate stereotypes, each employing only the variables that were significant or trending in Table 2 in order to preserve degrees of freedom. There was no effect of stereotypes about the out-group’s cooperativeness on participant giving to out-group strangers (−B0.02, p = 0.96), or even on keeping more money for the self or giving more to in-group strangers, i.e., to avoid out-group giving (−B0.05, p = 0.99; −B0.16, p = 0.81; Supplementary Table 5).

### (P3) Need

Participants who had been ill in the last month were gave less money to out-group strangers than those who had not been ill (−B0.90, p < 0.01), although they did not give any more money to in-group strangers or keep any more for themselves than healthy participants (B0.54, p = 0.18; B0.91, p = 0.52). There was no effect of resource shortfalls – that is, lower than usual income or production in the last two months, food insecurity, and household dependency – on giving.

### (P3) Network support

Participants who could borrow money from two or more communities where they had lived previously, or from someone in the local market town, gave more to *in-*group strangers than those who could not borrow from these other communities (B1.05, p < 0.05); they instead kept less for themselves, although the effect was not significant (−B2.20, p = 0.17). Those who engaged in cooperative labor exchanges in the past year gave more to out-group strangers than those who did not (B0.52, p < 0.05); a trend suggests they kept less money for themselves (−B1.87, p < 0.10; Fig. 2C).

### Exploratory analysis: Opting into anonymity

To ensure their autonomy, participants were given the option to play the game anonymously. We explored whether those who played non-anonymously behaved differently in the game than those who preferred to play anonymously. Revealing one’s name was unrelated to giving to out-group or in-group strangers, or to the amount kept for oneself. The effects for subjective socioeconomic status and recent illness were specific to participants who played non-anonymously, whereas the effect of number of places lived was robust across both samples (Supplementary Table 6). Whether one played anonymously or non-anonymously was included as a control variable in all models on the full sample.

### Additional variables

Other control variables did not have significant effects on giving (see Supplementary Table 2).

## Discussion

Little is understood about the social and ecological conditions that favor out-group relationship building in humans, though it is a hallmark feature of human sociality. Here we found that lowland Bolivian horticulturalists varying in their integration to Bolivian markets and society demonstrate substantial valuation for both in-group and out-group strangers, as measured by generosity in a non-anonymous economic game. We hypothesized that low non-local resource access and greater (positive) existing knowledge about out-groups should be associated with more generosity displayed toward an out-group stranger, while greater need and poor support networks should not differentially affect out-group and in-group giving. Overall, our results partially support our hypotheses. Consistent with P1, generosity toward out-group strangers was higher among those with minimal non-local resource access (i.e., lower subjective socioeconomic statuses relative to others in their community). Among the Tsimane’, but not the Mosetén or Interculturales, those who owned fewer market items were more generous toward out-group strangers. These associations between poor non-local resource access and out-group valuation suggest that an actor who can stand to benefit from resources best obtained non-locally (e.g., at markets) will consider new social partnerships, investing in her reputation as a reliable partner accordingly^12^,^27^,^30^.

We predicted that information about out-group members, particularly positive information about their potential cooperativeness, would modulate valuation for them (P2). In general, sources of information about out-group members increased out-group valuation, such as living in a greater number of other communities or watching more TV or movies. However, perhaps the most parsimonious explanation for these effects is the relationship between cosmopolitanism and out-group cooperation^34^. It may be those that have the highest levels of passive exposure to out-group members who value them the most highly, rather than those who simply have positive or negative information about them^35^. For example, holding negative stereotypes about the cooperative potential of an out-group – that is, having negative information about them – had no effect on giving.

Finally, our proposal that greater need or a lack of social support should not differentially affect valuation for out-group and in-group members (P3) was not supported. Participants who experienced a recent illness were less generous toward out-group strangers, but not in-group strangers, although our composite measure of recent resource shortfalls (including recent lower-than-expected production or income, food insecurity, and high dependency) bore no relationship to giving behavior. In terms of social support, participants who had cooperative partners for traditional cooperative labor gave more to out-group strangers, while those who could borrow from members of two other communities gave more to *in*-group strangers.

Our results contrast with existing studies that show greater market penetration increases giving to strangers, as market norms may prescribe behavior toward strangers^45^,^46^ or increased resource access may lower between-group competition by taking care of basic needs^10^. Instead, we find that higher subjective socioeconomic status, a measure of one’s perceived access to money and market items, predicts less generosity toward strangers that are members of out-groups, but not towards strangers from in-groups. However, our non-anonymous game substantially alters the game context (e.g.,^47^,^48^), so our results are not directly comparable. In fact, two commonly employed measures of market penetration (market asset value and household income) were largely unrelated to giving to out-group and in-group strangers. One reason the effect of market items may differ across populations is the different experiences of members of each population as they are exposed to out-groups; this may explain why the subjective measure of resource access, rather than the objective measures, had an effect in the full sample. For example, Tsimane’ participants may experience positive exposure to out-group members via the media but negative exposure via market interactions, as some Tsimane’ individuals suffer discrimination in local towns.

Likewise, the varied predictive power of different proxies for need and existing network connections provides insight into how out-group valuation may be modulated. For example, the negative relationship we found between recent illness and out-group relationships may be attributable to risk. Because out-group partnerships may be risky or initially costly – because norm systems between groups may differ, increasing transaction costs^18^, or because friends that rarely interact are more difficult to monitor (e.g.,^27^) – out-group valuation may become part of an actor’s risk management portfolio once some needs are met, and not when resources are especially scarce (e.g.,^15^,^23^,^49^). Participants who could borrow money from members of other communities likewise may have invested more in in-group members because their relative gains from additional sources of non-local resource access were lower relative to the costs of risky initial out-group interactions. These explanations are all consistent with an evolved psychology sensitive to the relative benefits and costs of out-group relationships^12^,^27^. Our future work will employ experimental manipulations and sources of quasi-natural experiments in Bolivia to better understand why some factors had effects here and not others, and which factors weigh the most heavily in out-group valuation.

In conclusion, the idea that out-group valuation may enable non-local resource access and buffering is consistent with existing ethnographic, archaeological, and social psychological data. The human foraging ecology is unique, based on the acquisition of calorically dense, difficult-to-acquire foods. Local inter-generational and intra-generational resource transfers buffer some of the variance in acquisition; however, our success has been partially dependent on maintaining social network connections at a distance from our local communities, as these connections provided crucial access to resources^9^,^44^, mates^13^, and information^50^,^51^. Further, as we suggest here, relationships with individuals from different groups – ethnic groups, groups with different modes of production, and in the contemporary word, religious groups – were also likely important sources of resource access^16^, mates, and information^14^, particularly for non-local resources^14^,^15^. Indeed, humans are strategically parochial, sensitive to the potential benefits to be gained via out-group relationships^11^,^29^,^35^,^49^. We suggest that evolutionary selective forces have favored a psychology sensitive to the relative benefits and costs of out-group relationships, especially the relative benefits in the currency of non-local resource access. This psychology is still at work in contemporary urban settings, where we interact with an unprecedented number of strangers. Understanding the relevant cues that increase the likelihood of out-group valuation, such as a desire for non-locally available resources, as was our focus here, should provide insight into how to address a variety of social ills, including cronyism and discrimination-based civil conflict.

## Methods

### Study populations

The Tsimane’, the Mosetén, and the multicultural community here called “Intercultural” are three populations of South American horticulturalists in the Bolivian lowlands. Together, members of the three populations capture the range of variation in market integration among lowland Bolivian horticulturalists. For example, the Tsimane’, Mosetén, and Intercultural households in this sample have median incomes of $36, $260 and $323 per month, respectively (the national private sector median is $560^52^). By interviewing members of all three populations, we measured a broad range of variation in exposure to out-group members and non-local market access. For additional ethnographic details, see Supplementary Methods 1.

“Groups” relevant in the Bolivian context are clusters of individuals who self-identify as the same ethnicity, religion, political party, work cooperative, or labor union. For this study, we identified non-political groups which members of our study populations could join or, in the case of ethnic groups, with whom they could potentially interact; focus is on religious and ethnic groups, as they are large enough to contain strangers. Mosetén participants regularly interact with members of six ethnic groups and may become members of two local work cooperatives. The majority of Mosetenes are Catholic, but an Evangelical Friends congregation is also part of the community. Intercultural has four churches and three local cooperatives. We selected five focal ethnic groups of the eight with whom Interculturales regularly interact. In their language and in conversation, the Tsimane’ distinguish between three native lowland ethnic groups (the Mosetén, Yuracaré, and Trinitarios) but cognize Andean immigrants to the lowlands as one group (*collas*) and non-indigenous lowlanders as another (*cambas*). Three churches have an intermittent presence in Tsimane’ communities, but Tsimane’ participants were not part of any work cooperatives.

### Experimental and survey protocol

150 adults age 18+ (male = 54%; 31% Tsimane, 35% Mosetén, 34% Intercultural) sampled from the three study populations were interviewed between August 2014–March 2015. Among the Tsimane’ and Mosetén, we sampled one adult from every household in the study communities; among the Interculturales, we sampled one adult from every household in four of the six neighborhoods of the community. As literacy is variable among these populations, participants gave their informed verbal consent to participate. Study protocol was approved by the University of California, Santa Barbara Institutional Review Board and research was carried out in accordance with the approved guidelines. Order of presentation of survey sections and items within sections were counterbalanced across participants. Protocols were developed based on pilot and ethnographic interviews conducted in each of the three study populations. See Supplementary Methods 2 for details about sampling and comprehension checks for the economic game.

### In-groups and out-groups

Participants sorted cards representing local groups on a physical five point scale, which drew on notions linguistic concepts of social closeness in Spanish and the Tsimane’ language (Supplementary Fig. 1); cards in the closest square were 1 = “groups I belong to most or feel most a part of,” while groups in the farthest were 5 = “groups I belong to least.” Cards placed in positions 1 and 2 were classified as “in-group” and those from positions 4 and 5 as “out-group”; from these, two groups were selected for the game: either one ethnic in-group and one ethnic out-group, or one religious in-group and one religious out-group.

### Generosity

A non-anonymous economic game loosely based on the Dictator Game^53^ and Allocation Game^47^ was designed to measure participants’ cooperative intent toward strangers. Photos of six candidate same-sex recipients–three in-group strangers and three out-group strangers within ten years of her age–were arrayed on the table (Fig. 1). All were past participants in the experiment (members of the pilot study had their photos taken, but did not play the game), and we ascertained that all were strangers before proceeding. Participants were told the name, group affiliation, and age (an intended distractor) of each individual. Stacks of three one boliviano coins (Bs; US $0.14 = B1) were placed on each photo and in front of the participant (total stake of B21; approximately 1/3 of a day’s wage). A participant could move any number of coins between photos, from photos to her own stack, or from her stack to the photos. Participants were informed that any Bs left on a photo would be given to that person in the participant’s name (unless the participant wished to remain anonymous) and any Bs left in front of the participant would be hers. To avoid confusion and maintain participants’ trust, donors who kept money for themselves received their payouts at the end of the interview, while recipients were given their payouts, along with the names of the donors and the amounts given, at the end of the field season. (We control for time preferences in all models with one potential proxy; see Supplementary Methods 5 and 6.) For analysis, we averaged the amounts a participant gave to the three out-group members and, for comparison, to the three in-group members. There was no difference in the amount of money allocated to members of out-group religions vs. members of out-group ethnic groups, so we combine both group types in all analyses.

### Existing non-local resource access

Aspects of participants’ access to market goods and money, including subjective socioeconomic status relative to others in their community^54^, were obtained by interview. Per previously evaluated methods used among the Tsimane’^38^, the population with the lowest levels of literacy of the three, household net income was calculated from participants’ self-reported earnings and expenditures on debts and wages over the last month. Participants also identified the quantity of popular market possessions owned by their household (Supplementary Methods 3). 2015 price was used to ascribe dollar amounts to market possessions. Because a participant’s comparison of herself to others may affect whether she believes she can access more resources elsewhere^55^, net income and dollar amount invested in market possessions were converted to z-scores at the sample level (i.e., all three populations together). Subjective socioeconomic status was logged to normalize the positively skewed data.

### Past exposure

Participants listed the number of towns and cities they had visited in their lifetime, as well as the number of locations where they had lived; number of locations lived and visited were each summed. They also indicated the number of hours they spent watching TV or movies in the past week. Due to the highly variable distributions of cities or towns visited and hours of TV or movies watched, these two variables were converted to z-scores at the sample level.

### Stereotypes

Participants were asked to describe what other people said about the focal out-group. These free-responses were later coded by whether they concerned the presence of traits related to cooperation (or of traits that would reduce the likelihood of cooperative outcomes). These codes were lumped into a binary variable: whether a participant had mentioned a negative stereotype with regard to cooperation, or had not. For examples of participant responses coded as negative stereotypes, see Supplementary Table 7.

### Need

Participants’ access to sufficient household resources was measured as self-reported food insecurity^56^ (Supplementary Methods 4), personal illness lasting three days or more in the past month, dependency (number of children living in the home), and whether production and earnings during the previous month were the same, higher, or lower than normative for the household. A summary measure of food insecurity, dependency, and changes in income/production was constructed by including all three in a principal components analysis and extracting the first component (variance explained = 47.03%). Recent illness did not load on this component and is thus considered separately.

### Network support

To capture whether participants had existing social connections that could buffer shortfalls affecting their local community, participants were asked whether they could stay with a family from a different community (i.e., in their natal community, a community where they had lived previously, or in the local market town) during a hypothetical flood affecting their household, and whether they could ask someone from these communities for a loan of B100 (1.5 to 2 days’ wages). Additionally, the limited availability of trustworthy cooperative partners constrains the ability to engage in traditional cooperative labor in these populations: ethnographic interviews indicate that many abandon cooperative labor when it is not reciprocated, suggesting a lack of cooperative labor reflects a lack of support from one’s network connections. As such, participants were also asked if they gave or received cooperative labor in the previous year.

### Additional variables

We controlled for other factors that might affect out-group valuation beyond the scope of the present hypotheses (Supplementary Methods 5). These include participants’ propensity toward risk taking (Supplementary Methods 6) and their Agreeableness (Supplementary Methods 7), which may independently predict prosocial tendencies in the economic game^57^ and associate with past exposure and network support^58^. We also considered whether a participant preferred to remain anonymous in the economic game, as participants may play differently if they believe their decisions are anonymous^48^.

### Statistical methods

All analyses were performed using the R statistical program^59^. We compared the predictors of generosity toward out-group vs. in-group strangers vs. money kept by the participant across models. Because generosity data violated Gaussian distributional assumptions, all models use a Bayesian approach (MCMCglmm)^60^. Model estimates are reported as means of the posterior distribution. Unless specified otherwise, all models include data from all three populations, population random intercepts, and control for survey version (i.e., six alternative orders of interview sections and questions within these sections). Data are available in Supplementary Data 1, and are described in Supplementary Methods 8.

Collinearity was assessed for each model with a maximum permissible variance inflation factor of 4. Highly correlated variables were not included in the same models. To check the robusticity of our effects, we fit each model within each population. To avoid the influence of outliers, we rounded extreme values and transformed non-normal continuous predictors (Supplementary Table 8).

The code used to conduct analyses is available in the Supplementary txt file.

## Additional Information

**How to cite this article**: Pisor, A. C. and Gurven, M. Risk buffering and resource access shape valuation of out-group strangers. *Sci. Rep.* **6**, 30435; doi: 10.1038/srep30435 (2016).

## Supplementary Material

Supplementary Information

Supplementary Dataset 1

Supplementary Text File

## Figures and Tables

**Table 1:** Summary of results for valuation of out-group and in-group strangers.

Variable	Out-group		In-group	
	Prediction	Result	Prediction	Result
***(P1) Existing non-local resource access***	↓		N.E.	
*Income last month*		N.E.		N.E.
*Value of market items*		N.E.^1^		N.E.
*log subjective SES*		↓		N.E.
***(P2) Past exposure***	↑		N.E.	
*Hours TV/movies*		↑		N.E.
*Cities/towns visited*		N.E.		N.E.
*Places lived*		↑		N.E.
*No negative out-group stereotype*		N.E.		N.E.
***(P3) Need***	↑		↑	
*Shortfall summary*		N.E.		N.E.
*Recent illness*		↓		N.E.
***(P3) Network support***	↓		↓	
*Can borrow from 1 comm.*		N.E.		N.E.
*Can borrow from 2*+ *comms.*		N.E.		↑
*Can stay in other comm.*		N.E.		N.E.
*No traditional labor*		↓		N.E.

All effects for which p < 0.10 are reported. N.E. signifies no effect. Estimates for out-group stereotypes appear in Supplementary Table 5. ^1^Value of market items has a significant negative effect on out-group giving, but only among the Tsimane’.

**Table 2:** Estimates for the effects of each predictor on out-group and in-group giving, respectively.

Variable	Out-group				In-group			
	Post. mean	Lower 95%	Upper 95%	p value	Post. mean	Lower 95%	Upper 95%	p value
*Intercept*	2.13	−0.32	4.50	0.07	0.06	−2.29	2.37	0.96
***(P1) Existing non-local resource access***								
*Income last month*^◊^	0.06	−0.18	0.30	0.65	0.14	−0.18	0.47	0.40
*Value of market items*^◊^	−0.03	−0.31	0.27	0.85	0.17	−0.17	0.53	0.32
*log subjective SES*	−0.33	−0.70	0.01	0.07	0.14	−0.29	0.59	0.52
***(P2) Past exposure***								
*Hours TV/movies*^◊^	0.23	−0.05	0.49	0.10	0.03	−0.28	0.35	0.83
*Cities/towns visited*^◊^	0.09	−0.17	0.38	0.50	−0.22	−0.55	0.11	0.19
*Places lived*	0.21	0.05	0.36	0.01	−0.03	−0.22	0.15	0.73
***(P3) Need***								
*Shortfall summary*	−0.05	−0.25	0.17	0.67	−0.13	−0.38	0.13	0.33
*Recent illness*	−0.90	−1.52	−0.26	0.00	0.54	−0.24	1.35	0.18
***(P3) Network support***								
*Can borrow from 1 comm.*	−0.30	−1.10	0.47	0.45	0.31	−0.59	1.21	0.51
*Can borrow from 2*+ *comms.*	−0.42	−1.18	0.30	0.26	1.05	0.13	1.89	0.02
*Can stay in other comm.*	−0.05	−0.58	0.49	0.85	0.30	−0.34	0.96	0.37
*Traditional labor partners*	0.52	0.01	1.01	0.04	0.19	−0.38	0.80	0.52

Out-group model sample size = 150, DIC = 532.17; in-group model sample size = 133, DIC = 513.60. Estimates are means of the posterior distribution (fit with MCMCglmm). Population is included as a random effect in all models. Estimates for control variables (playing non-anonymously, risk proneness, Agreeableness, Extraversion, sex, age, marital status, years of schooling, and times attended church in the last month) are reported in Supplementary Table 2. (Survey version is included as a control in all models but not reported.) No variables evidenced collinearity (i.e., all exhibited a variance inflation factor of less than 4). ^◊^Variables reflect z-scores, e.g., a participant’s household income in the last month relative to the mean household income in the full sample.
