# Supplementary material for: Risk buffering and resource access shape valuation of out-group strangers
Source: Sci Rep. 2016 Jul 29;6:30435. doi: 10.1038/srep30435 (PMC4965756; doi:10.1038/srep30435)
Supplement: Supplementary Information [file srep30435-s1.pdf]

Supplementary information: Risk-buffering and resource access shape valuation of out-group strangers

Anne C. Pisor, Michael Gurven

Table of Contents

Supplementary Methods 1. Additional ethnographic details

Supplementary Methods 2. Additional details about sampling and checking game comprehension

Supplementary Methods 3. List of candidate market possessions by population

Supplementary Methods 4. Adapted food security questionnaire

Supplementary Methods 5. Additional variables included in analyses

Supplementary Methods 6. Adapted risk taking questionnaire

Supplementary Methods 7. Adapted personality inventories

Supplementary Methods 8. Description of supplemental data

Supplementary Table 1. Descriptive statistics on the full sample

Supplementary Table 2. Parameter estimates for out-group and in-group giving, including controls

Supplementary Table 3. Parameter estimates for money kept for the self, including controls

Supplementary Table 4. Parameter estimates for out-group giving by population, including controls

Supplementary Table 5. Parameter estimates for out-group giving, in-group giving, and money kept for the self for the subsample for which stereotype data were available

Supplementary Table 6. Parameter estimates for out-group giving by whether the participant wished to share their name or remain anonymous, including controls

Supplementary Table 7. Examples of responses coded as negative or positive stereotypes about cooperative potential

Supplementary Table 8. Adjustments for outliers and heteroscedasticity

Supplementary Figure 1. Card sort methodology

Supplementary Methods 1 – Additional ethnographic details

The Mosetén and Tsimane' are two of the 36 *pueblos indígenas* recognized by the Plurinational State of Bolivia. The two populations have recognized territories (*Tierras Comunitarias de Origen*) on either side of a range of mountains separating the Beni Department from the La Paz Department of Bolivia. Historically the two populations intermarried and had highly similar languages and practices – together, the Mosetén and Tsimane' languages are a linguistic isolate known as Mosetenan [1] – but the Mosetén were missionized a century earlier [2]. Catholic Franciscan clergy established schools for the Mosetén and, by the end of the 20th century, had helped fund the paving of roads connecting the area to the market town, enabling extensive cash cropping and logging over the last three decades. The Mosetén have increasingly intermarried with non-Mosetén (Quechua and Aymara speakers), who then settled in the Mosetén territory; approximately half of all households in the communities sampled include one non-Mosetén adult member. While all Mosetenes speak fluent Spanish, the dominant language in Bolivia, only 14% of Tsimane' participants speak Spanish fluently. Evangelical New Tribes and Catholic Redemptorist missionaries began work with the Tsimane' in the early 1950s. Dirt roads were constructed in 1975 but have reached few communities; many Tsimane' continue to rely on river travel, which has curbed market penetration. Today the majority of Tsimane' communities have schools, but roughly one third were constructed only in the last decade. The Tsimane' remain predominantly endogamous: when intermarriage does occur, the Tsimane' tend to marry lowland ethnic groups living in or near Tsimane' territory. Poor preservation in the region limits our ability to estimate how long the Mosetén and Tsimane' have lived in this area [3], but haplotype data suggest that they are more closely related to populations in the Andes than other lowland populations [4].

A third population is a multicultural (*intercultural*) community located at the boundary of Mosetén territory in the Beni River Valley (the community is here called "Intercultural"). The Mosetén historically lived in the area, joined in the mid-20th century by Trinitarios searching for the promised land

(*la tierra santa*). In the 1960s, a government-sponsored colonization project relocated Aymara speakers from the Andes to the Intercultural area. In response, most Mosetén and Trinitario families moved farther upriver. Many Aymara speakers ultimately returned to the Andes, but a small consortium of families who remained in the area – Aymaras, Trinitarios, and immigrants from the Beni – secured funds for a road to the local market town in 1975 and founded Intercultural in 1979. In the last four decades, Intercultural has grown to a population of 1100 as residents have immigrated both from nearby communities and distant regions for the favorable growing climate and soils or the now-dwindling logging industry. Intercultural participants were predominantly Aymara (59%) and Quechua (18%), the two most populous indigenous groups in Bolivia.

Supplementary Methods 2 – Additional details about sampling and checking game comprehension

Tsimane' interviews were conducted in the Tsimane' language with the help of a research assistant, Mosetén and Interculturales interviews in Spanish by the researcher. Tsimane' interviews were translated from Spanish to Tsimane' and back-translated until concepts were conveyed consistently in both languages. Interviews were conducted in two parts across two different days. An attempt was made to sample one adult from each household in each community, with an equal number of male and female participants. Individuals who engaged in more wage labor or sold more produce may have been less likely to be at home than other members of the community; to compensate for this, households whose members were absent were re-visited at regular intervals in an attempt to interview these individuals. Instructions for the economic game were given at the beginning of the second interview using example individual recipients (cartoon faces named Juan, Carlos, and Patricia) and U.S. pennies in place of *bolivianos* (*Bs*). To ascertain participant understanding, two possible allocations were demonstrated and participants were asked to tell the researcher how much each group or individual would receive in each circumstance. The researcher proceeded only if a participant correctly reported

the amounts; if they did not, the researcher repeated the instructions for the experiment and demonstrated a third allocation. Participants were compensated with household gifts equivalent in value to one hour's wages for each interview.

Supplementary Methods 3 – Popular market possessions as assessed during pilot ethnographic research

- Mosetén and Interculturales: Cars/trucks, TVs, satellites, stores or small restaurants, refrigerators or freezers, cell phones, chainsaws, string trimmers.
- Tsimane': Outboard motors, TVs, cell phones, chainsaws.

Supplementary Methods 4 – Food security questionnaire as adapted from [5] for use among Bolivian horticulturalists

Scoring: Responding "always" to a given question was given a score of 1, "never" a score of 0, and sometimes a score of 0.5. Scores were summed across questions.

1. In the last 12 months, how frequently did you think the food in your household would run out before you could get more? Always, sometimes, or never?

(Spanish) *En los últimos 12 meses, con qué frecuencia pensabas que la comida/los viveres de tu casa va a acabar antes de podrías sacar más? Siempre, a veces, o nunca?*

(Tsimane') *Oij yomodye' jiyaques, jun buty quim' ca dyijyim paj qui moyam jibitidyes aca'yadyes mi aty räi' ya, janas qui buty daque mdyijyica' mi mo'ra jibitidyes mi a aty jam bura' jam dami? Räjcan, are' jämdye'ya', are' jam jam yiri'?*

2. In the last 12 months, how frequently did the food in your household run out and you could not get more? Always, sometimes, or never?

En los últimos 12 meses, con qué frecuencia acabó la comida/los viveres de tu casa y no podías sacar más? Siempre, a veces, o nunca?

Oij yomodye' jiyaques, jun buty quim' aty räi' jibitidyes aca'ya'dyes mi, aty jam jun buyi quim daĉan dam dyem? Räjcan, are' jämdye'ya', are' jam jam yiri'?

3. In the last 12 months, how frequently could you not provide a complete diet for your household? Always, sometimes, or never?

En los últimos 12 meses, con qué frecuencia no podías sacar comida completa/viveres completos para tu casa?

Oij yómodye' jiyaques, aty buty quim jam cuts jibiti'dyes aca'ya'dyes mi? Räjcan, are' jämdye'ya', are' jam jam yiri'?

4. In the last 12 months, how frequently did your household only have a few kinds of inexpensive food or food that was not tasty because you could not get more? Always, sometimes, or never?

En los últimos 12 meses, con qué frecuencia tenía tu casa solamente unos tipos de comida/viveres barata o comida/viveres de mal sabor porque no podías sacar más? Siempre había comida buena? Siempre, a veces, o nunca?

Oij yomodye' jiyaques, jedye ca momo mo'ya aca'ya' mi, are' mo' dyi' momo' jibitidyes mi? Räjcan, are' jämdye'ya', are' jam jam yiri'?

Supplementary Methods 5 – Additional variables included in analyses

Actors more prone to risk may be more likely to engage in potentially costly initial generosity toward out-groups [6], but those who discount the future may invest more in the self or in existing relationships rather than new ones [7]; as such, we used four questions to measure participants' risk proneness, one of which also gauges temporal discounting ([8]; Supplementary Methods 6). We also controlled for

Agreeableness, which is a predictor of prosocial behavior in economic games[9], and Extraversion, which increases likelihood of exploration and forming new social relationships[10] (see Supplementary Methods 7). We controlled for frequency of church attendance in the past month, as attendance could increase in-group favoritism [11] or increase the likelihood that participants felt their actions might be observed by an omniscient god [12]. Sex differences in generosity and cooperativeness are prevalent [13] and cohort effects on trust have been reported [14], so we included both sex and age in all models. Education increases exposure to information about out-groups, whether positive or negative in content; also, participants seeking additional resources through out-groups may build their human capital to attain access. Because of these possibilities, we controlled for a participant's highest level of schooling. Mate search may increase out-group exposure, although it is not consistently associated with marital status in the Bolivian context; we include marital status as an imperfect potential predictor of out-group valuation.

Supplementary Methods 6 – Stimulating and instrumental risk taking questionnaire adapted from [8] for brevity and use among Bolivian horticulturalists

Scoring: Items were scored on a five point scale, with "true" responses scored as 5 and "false" scored as 1. Scores were summed across questions.

1. You take risks only if it is necessary to achieve something. True, almost true, somewhat true, almost false, or false?

(Spanish) *Tomas riesgo solamente si es necesaria para lograr algo. Verdadero, casi verdadero, intermedio, casi falso, o falso?*

(Tsimane') *Me tsan anic carij midyes miqui nac anic jemoñe si' mi tupuj me'jetaque' mi. Anic me' o me' o dam' momo o jam o jam yirity?*

2. You do not like to do things whose results depend too much on chance. If something depends a lot on chance, you do not do it. True, almost true, somewhat true, almost false, or false?

No quieres hacer cosas cuyos resultados dependen demasiado mucho en la suerte. Si algo depende mucho en la suerte, no lo haces. Verdadero, casi verdadero, intermedio, casi falso, o falso?

Tupuj dyäcje mi mo carijtaqui mi jam juiya mujucha tuyin bijodye jeñej mo' in cätidye in metsam mo'ya' tupudye jam qui nac jämi' ya in. Anic me' o me' o dam' momo o jam o jam yirity?

3. Do you prefer work for a larger wage that can end any day more than stable work with a smaller wage, for example, contract work that does not pay you well? True, almost true, somewhat true, almost false, or false?

Prefieres más un trabajo por un sueldo más grande que se puede terminar cualquier día o un trabajo estable con un sueldo más pequeño, por ejemplo, un trabajo con contracto que no te paga bien? El primero, el segundo, o los dos por igual?

Ma'je' buty mi yiris carijtacdye yirity tum yaitacdye jämtyi, jam cavin räi'si' mo tacya chime moya carijtacdye damsi yaitacdye, ejemplo carijtacdye contrato in?

4. To achieve in life, you need to take risks. True, almost true, somewhat true, almost false, or false?

Para lograr algo en esta vida, necesitas tomar riesgos. Verdadero, casi verdadero, intermedio, casi falso, o falso?

Paj qui jäm' joij ve juijya' mi jemoñe buty mi me'je metsan' carij midyes. Anic me' o me' o dam' momo o jam o jam yirity?

Supplementary Methods 7. Personality inventories adapted from [15] for brevity and use among Bolivian horticulturalists

Scoring: Items were scored on a five point scale, with "true" responses scored as 5 and "false" scored as 1. Scores were summed across questions.

- **Agreeableness**

1. You do not want to help other people if helping them will disadvantage you. If it will cost you, you will not help others. True, almost true, somewhat true, almost false, or false?

No quieres ayudar a otras personas si esa ayuda lleva desventajas para ti. Si hay un gasto para ti, no vas a ayudarles. Verdadero, casi verdadero, intermedio, casi falso, o falso?

Jam adac ma'je notacsi yoctyi muntyi in, mo qui nam notacdye' mi jam jäm juijya midyes. Anic me' o me' o dam' momo o jam o jam yirity?

2. You like to be generous without expecting a service in return. True, almost true, somewhat true, almost false, or false?

Te gusta ser generoso sin esperar un servicio a cambio. Verdadero, casi verdadero, intermedio, casi falso, o falso?

Ma'je buty mi pajqui so'macsity jedye' mi, jam bisem yoctyis notacdye. Anic me' o me' o dam' momo o jam o jam yirity?

3. Your well-being is more important to you than the problems of other people. True, almost true, somewhat true, almost false, or false?

Tu bienestar es más importante a ti como los problemas de otras personas. Verdadero, casi verdadero, intermedio, casi falso, o falso?

Mo' jäm'jodye' anic buty jemonac cui dyes midyes mi, jam jeñej mu in carijsistumtyi' yoctyi muntyi' in. Anic me' o me' o dam' momo o jam o jam yirity?

4. You would help other people even if you have serious problems of your own. True, almost true, somewhat true, almost false, or false?

Todavía ayudes a otra gente aunque tienes tus propios problemas graves. Verdadero, casi verdadero, intermedio, casi falso, o falso?

Taca' buty nótacsi yoctyi' muntyi' mi, me'tsan' mis mo'ya' cui'si' carijsis anic are'sis. Anic me' o me' o dam' momo o jam o jam yirity?

- **Extraversion**

1. When you are together with a lot of people, you prefer to be apart from them. True, almost true, somewhat true, almost false, or false?

Cuando tú estás junto a muchas personas prefieres que quedarte fuera de ellos. Verdadero, casi verdadero, intermedio, casi falso, o falso?

Me' juijya mi mu'ya yiri'ya daityiya muntyi' in, tupuj buty jäquive bu'yi mi jorajyayeban mu in ya in. Anic me' o me' o dam' momo o jam o jam yirity?

2. You feel better when there are a lot of people around you. True, almost true, somewhat true, almost false, or false?

Sientes mejor cuando hay muchas personas cerca de ti. Verdadero, casi verdadero, intermedio, casi falso, o falso?

Jäm' cuti mi me' mu'ya' dai' muntyi' in tyeijya juijya in. Anic me' o me' o dam' momo o jam o jam yirity?

3. You feel better when you are alone. True, almost true, somewhat true, almost false, or false?

Sientes mejor cuando estás solo. Verdadero, casi verdadero, intermedio, casi falso, o falso?

Jämyi buty mi yirity dyety juijyam. Anic me' o me' o dam' momo o jam o jam yirity?

4. You always have fun meeting new people. True, almost true, somewhat true, almost false, or false?

Siempre te diviertes a conocer personas nuevas. Verdadero, casi verdadero, intermedio, casi falso, o falso?

Räjĉan buty ma'jotacsi mi quimdyem atysijtyi' muntyi' in. Anic me' o me' o dam' momo o jam o jam yirity?

Supplementary Methods 8 – Description of supplemental data

A csv file of the data used in these analyses is available as part of the supplementary materials. To protect participant identity, participant PIDs are false, community names are excluded, and participant ages are rounded to the nearest decade (e.g., ages 25-34 appear as age 30). Outliers have not been removed, except for variables included in the shortfall summary variable as was necessary to calculate these values (i.e., number of children, food security; see Table S1 for details about outlier removal).

Supplementary Table 1. Descriptive statistics on the full sample.

Variable	Mean	SD	Median	Min	Max	N□	% level 1	% level 2	% level 3	Level descriptions
Avg. to out-group	2.21	1.50	2.5	0	7	150				
Avg. to in-group	2.90	1.55	3	0	7	133				
Money kept	5.71	5.90	3	0	21	150				
(P1) Existing non-local resource access										
Income last month◇	-0.03	1.00	-0.32	-2.40	3.44	150				
Value of market items◇	0.04	1.00	-0.28	-1.13	2.27	150				
log subjective SES	1.02	0.70	1.10	0.00	2.30	150				
(P2) Past exposure										
Hours TV/movies◇	0.03	0.98	-0.30	-0.78	3.15	150				
Cities/towns visited◇	0.04	1.01	-0.34	-1.01	3.14	150				
Places lived	3.19	1.95	3	0	8	150				
No negative stereotypes	---	---	1	---	---	43	0.53	0.47		1=stereotype, 2=no stereotype
(P3) Need										
Shortfall summary	0.03	1.27	-0.04	-2.45	2.86	150				
Food insecurity+	1.40	0.87	1.5	0	3	150				
No. children in home+	2.67	1.92	3	0	7	150				
Produce/income below normal+	---	---	2	---	---	150	0.37	0.48	0.15	1=no shortage, 2=shortage of one thing, 3=shortage of two things
Recent illness	---	---	1	---	---	150	0.81	0.19		1=no illness this month, 2=ill this month
(P3) Network support										
Can borrow from other comms.	---	---	2	---	---	150	0.15	0.39	0.45	1=not from one, 2=from one, 3=from two
Can stay in other comm.	---	---	2	---	---	150	0.29	0.71		1=no, 2=yes

Traditional labor partners	---	---	2	---	---	150	0.39	0.61		1=no labor, 2=labor
Additional variables										
Share name with recipient	---	---	2	---	---	150	0.33	0.67		1=no, 2=yes
Risk proneness	7.60	2.83	8	0	12	150				
Agreeableness	7.55	3.26	8	0	16	150				
Extraversion	6.15	2.50	6	0	13	150				
Sex: Male	---	---	2	---	---	150	0.46	0.54		1=female, 2=male
Age	39.49	13.62	37.5	18	85	150				
Married	---	---	2	---	---	217	0.15	0.85		1=single, 2=married
Years schooling	6.82	4.51	7	0	16	150				
Times to church/mo.	1.97	1.80	2	0	5	150				
Population	---	---	2	---	---	150	0.31	0.35	0.34	1=Tsimane’, 2=Mosetén, 3=Intercultural

◇Variables z-scored. +Variables which form the shortfall summary measure is constructed. □Includes participants presented with recipients from intermediate groups (members of groups in position 3; see Supplementary Figure 1) in the economic game, though these participants are excluded from analysis.

Supplementary Table 2. Parameter estimates for out-group and in-group giving, including controls.

**Variable**	**Out-group**				**In-group**			
	**Post. mean**	**Lower 95%**	**Upper 95%**	**p value**	**Post. mean**	**Lower 95%**	**Upper 95%**	**p value**
Intercept	2.13	-0.32	4.50	0.07	0.06	-2.29	2.37	0.96
(P1) Existing non-local resource access								
Income last month$^{\diamond}$	0.06	-0.18	0.30	0.65	0.14	-0.18	0.47	0.40
Value of market items$^{\diamond}$	-0.03	-0.31	0.27	0.85	0.17	-0.17	0.53	0.32
log subjective SES	-0.33	-0.70	0.01	0.07	0.14	-0.29	0.59	0.52
(P2) Past exposure								
Hours TV/movies$^{\diamond}$	0.23	-0.05	0.49	0.10	0.03	-0.28	0.35	0.83
Cities/towns visited$^{\diamond}$	0.09	-0.17	0.38	0.50	-0.22	-0.55	0.11	0.19
Places lived	0.21	0.05	0.36	0.01	-0.03	-0.22	0.15	0.73
(P3) Need								
Shortfall summary	-0.05	-0.25	0.17	0.67	-0.13	-0.38	0.13	0.33
Recent illness	-0.90	-1.52	-0.26	0.00	0.54	-0.24	1.35	0.18
(P3) Network support								
Can borrow from 1 comm.	-0.30	-1.10	0.47	0.45	0.31	-0.59	1.21	0.51
Can borrow from 2+ comms.	-0.42	-1.18	0.30	0.26	1.05	0.13	1.89	0.02
Can stay in other comm.	-0.05	-0.58	0.49	0.85	0.30	-0.34	0.96	0.37
Traditional labor partners	0.52	0.01	1.01	0.04	0.19	-0.38	0.80	0.52
Additional variables								
Share name with recipient	0.09	-0.41	0.59	0.74	0.48	-0.12	1.10	0.12
Risk proneness	0.00	-0.08	0.08	0.99	0.06	-0.04	0.16	0.25
Agreeableness	0.04	-0.04	0.12	0.34	0.04	-0.06	0.13	0.45
Extraversion	-0.02	-0.11	0.08	0.74	0.02	-0.09	0.14	0.72
Sex: Male	-0.25	-0.79	0.26	0.34	0.15	-0.50	0.78	0.64
Age^{+}	-0.01	-0.03	0.01	0.36	0.01	-0.01	0.04	0.27
Married	-0.02	-0.66	0.65	0.94	0.68	-0.16	1.56	0.12
Years schooling	-0.03	-0.10	0.04	0.41	0.01	-0.08	0.09	0.86
Times to church/mo.	0.04	-0.09	0.17	0.56	0.09	-0.07	0.24	0.26

Both models control for survey version, which was counterbalanced. Out-group sample size=150, effective sample size (i.e., number of samples from the posterior distribution)=27,000; DIC=532.17. In-group sample size=133, effective sample size=26,510; DIC=513.60. ◇Variables z-scored. +Age is centered at age 18.

Supplementary Table 3. Parameter estimates for money kept for the self, including controls.

	Self			
**Variable**	**Post. mean**	**Lower 95%**	**Upper 95%**	**p value**
Intercept	14.56	6.10	22.45	0.00
(P1) Existing non-local resource access				
Income last month[◇]	-0.45	-1.49	0.58	0.40
Value of market items[◇]	-0.43	-1.62	0.80	0.48
log subjective SES	0.60	-0.93	2.12	0.44
(P2) Past exposure				
Hours TV/movies[◇]	-1.06	-2.18	0.03	0.06
Cities/towns visited[◇]	0.17	-1.01	1.37	0.78
Places lived	-0.62	-1.27	0.01	0.06
(P3) Need				
Shortfall summary	0.54	-0.37	1.43	0.24
Recent illness	0.91	-1.85	3.63	0.52
(P3) Network support				
Can borrow from 1 community	-0.58	-3.84	2.73	0.73
Can borrow from 2+ communities	-2.20	-5.27	1.03	0.17
Can stay in other community	-0.91	-3.26	1.41	0.45
Traditional labor partners	-1.87	-4.08	0.19	0.08
Additional variables				
Share name with recipient	-1.69	-3.97	0.41	0.13
Risk proneness	-0.10	-0.44	0.26	0.58
Agreeableness	-0.23	-0.55	0.11	0.17
Extraversion	-0.03	-0.43	0.37	0.90
Sex: Male	0.69	-1.63	2.94	0.55
Age[+]	-0.03	-0.11	0.06	0.52
Married	-1.21	-4.09	1.67	0.40
Years schooling	0.02	-0.31	0.33	0.90
Times to church/mo.	-0.40	-0.96	0.17	0.16

Model controls for survey version, which was counterbalanced. Sample size=133, effective sample size=27,000; DIC=973.45. ◇Variables z-scored.

+Age is centered at age 18.

Supplementary Table 4. Parameter estimates for out-group giving by population, including controls.

	Tsimane'				Mosetén				Interculturales			
**Variable**	**Post. mean**	**Lower 95%**	**Upper 95%**	**p value**	**Post. mean**	**Lower 95%**	**Upper 95%**	**p value**	**Post. mean**	**Lower 95%**	**Upper 95%**	**p value**
Intercept	1.77	-3.00	6.48	0.45	1.91	-0.65	4.64	0.15	1.50	-1.97	4.82	0.38
(P1) Existing non-local resource access												
Income last month◇	0.10	-0.96	1.16	0.85	0.12	-0.28	0.51	0.54	-0.12	-0.50	0.27	0.55
Value of market items◇	-2.72	-4.56	-0.92	0.00	0.12	-0.33	0.56	0.59	-0.08	-0.54	0.35	0.70
log subjective SES	-0.28	-1.15	0.62	0.53	-0.47	-1.07	0.16	0.13	-0.05	-0.86	0.73	0.90
(P2) Past exposure												
Hours TV/movies◇	3.45	0.38	6.62	0.03	0.26	-0.12	0.66	0.19	0.22	-0.21	0.62	0.30
Cities/towns visited◇	0.48	-2.82	3.79	0.77	0.16	-0.26	0.61	0.45	-0.17	-0.61	0.29	0.44
Places lived	-0.01	-0.53	0.49	0.97	0.20	-0.03	0.43	0.09	0.15	-0.14	0.43	0.31
(P3) Need												
Shortfall summary	0.00	-0.64	0.68	1.00	-0.07	-0.39	0.24	0.64	-0.23	-0.63	0.18	0.26
Recent illness	---	---	---	---	-0.38	-1.28	0.51	0.38	---	---	---	---
(P3) Network support												
Can borrow from 1 comm.	-0.46	-1.97	0.95	0.52	0.81	0.09	1.51	0.02	-0.37	-1.19	0.46	0.37

Can borrow from 2+ comms.	-0.66	-2.24	0.89	0.39	---	---	---	---	---	---	---	---
Can stay in other comm.	0.06	-1.25	1.32	0.92	0.20	-0.72	1.10	0.66	-0.45	-1.47	0.56	0.36
Traditional labor partners	1.03	-0.32	2.40	0.13	0.68	-0.17	1.53	0.11	0.28	-0.67	1.28	0.57
Additional variables												
Share name with recipient	1.44	-0.15	3.05	0.07	-0.12	-0.91	0.65	0.75	0.37	-0.65	1.36	0.47
Risk proneness	0.05	-0.16	0.27	0.63	0.00	-0.12	0.13	0.99	0.04	-0.12	0.21	0.60
Agreeableness	-0.04	-0.24	0.16	0.71	0.06	-0.07	0.20	0.34	0.09	-0.05	0.23	0.21
Extraversion	0.05	-0.20	0.29	0.69	-0.02	-0.16	0.13	0.80	0.04	-0.18	0.27	0.74
Sex: Male	-1.07	-2.53	0.39	0.15	0.27	-0.54	1.10	0.50	0.70	-0.59	1.96	0.27
Age[+]	0.02	-0.03	0.07	0.50	-0.01	-0.05	0.04	0.75	-0.01	-0.05	0.02	0.43
Married	---	---	---	---	---	---	---	---	-0.21	-1.20	0.84	0.69
Years schooling	-0.02	-0.29	0.24	0.89	-0.02	-0.12	0.09	0.75	-0.04	-0.16	0.08	0.51
Times to church/mo.	0.07	-0.22	0.38	0.63	-0.08	-0.31	0.18	0.53	0.26	-0.03	0.55	0.08

Categorical variables excluded when number of individuals at each level was too small in a given population. Survey version is excluded from all three models due to issues with collinearity. Being able to borrow from past communities is binned into two categories – no communities and one community in one bin, two or more communities in the other – for the Mosetén and Interculturales. Tsimane' sample size=47, effective sample size=27,000; DIC=199.31. Mosetén sample size=52, effective sample size=27,000; DIC=180.08. Intercultural sample size=51, effective sample size=27,403; DIC=190.16. [◊]Variables z-scored. [+]Age is centered at age 18.

Supplementary Table 5. Parameter estimates for out-group giving, in-group giving, and money kept for the self for the subsample for which stereotype data were available.

Variable	Out-group				In-group				Self			
	**Post. mean**	**Lower 95%**	**Upper 95%**	**p value**	**Post. mean**	**Lower 95%**	**Upper 95%**	**p value**	**Post. mean**	**Lower 95%**	**Upper 95%**	**p value**
Intercept	0.74	-0.85	2.36	0.34	4.47	2.31	6.71	0.00	4.50	-2.64	12.02	0.22
(P1) Existing non-local resource access												
log subjective SES	-0.68	-1.41	0.04	0.07	-0.57	-1.60	0.50	0.28	3.94	0.45	7.45	0.03
(P2) Past exposure												
Hours TV/movies◇	0.54	-0.10	1.19	0.10	-0.01	-0.87	0.88	0.97	-0.95	-3.88	1.89	0.51
Places lived	0.29	0.02	0.54	0.03	0.17	-0.20	0.54	0.36	-1.45	-2.66	-0.21	0.02
No negative out-group stereotype	-0.02	-0.93	0.86	0.96	-0.16	-1.50	1.15	0.81	-0.05	-4.39	4.22	0.99
(P3) Need												
Recent illness	-0.59	-1.55	0.33	0.21	1.35	-0.11	2.79	0.07	-2.52	-7.10	2.01	0.27
(P3) Network support												
Traditional labor partners	0.38	-0.46	1.23	0.37	0.10	-1.13	1.33	0.87	2.07	-1.93	6.27	0.31

Only variables which were significant in models reported above were included in these models, to preserve degrees of freedom given small sample sizes. Both models control for survey version, which was counter-balanced. Out-group sample size=42, effective sample size=26,331; DIC=152.10. In-group sample size=38, effective sample size=26,524; DIC=165.68. Money kept for self sample size=44, effective sample size=27,000, DIC=288.29. Out-group model including stereotypes provides a worse fit than model excluding stereotypes (DIC=152.10 vs DIC=149.71). ◇Variable z-scored.

Supplementary Table 6. Parameter estimates for out-group giving by whether the participant wished to share their name or remain anonymous, including controls.

	Non-anonymous				Anonymous			
**Variable**	**Post. mean**	**Lower 95%**	**Upper 95%**	**p value**	**Post. mean**	**Lower 95%**	**Upper 95%**	**p value**
Intercept	2.91	0.64	5.20	0.01	2.33	-1.04	5.70	0.17
(P1) Existing non-local resource access								
Income last month◇	0.02	-0.28	0.33	0.88	-0.17	-0.72	0.37	0.54
Value of market items◇	0.19	-0.17	0.56	0.30	0.16	-0.42	0.74	0.57
log subjective SES	-0.38	-0.80	0.06	0.08	-0.70	-1.55	0.14	0.10
(P2) Past exposure								
Hours TV/movies◇	0.13	-0.23	0.48	0.47	0.43	-0.18	1.05	0.17
Cities/towns visited◇	0.38	-0.01	0.76	0.06	-0.22	-0.75	0.30	0.39
Places lived	0.17	-0.02	0.37	0.08	0.41	0.12	0.69	0.01
(P3) Need								
Shortfall summary	-0.13	-0.39	0.14	0.33	-0.11	-0.65	0.45	0.70
Recent illness	-1.02	-1.82	-0.20	0.01	-0.40	-1.83	1.08	0.58
(P3) Network support								
Can borrow from 1 comm.	-0.62	-1.55	0.33	0.20	0.61	-0.42	1.70	0.24
Can borrow from 2+ comms.	-0.56	-1.45	0.33	0.21	---	---	---	---
Can stay in other comm.	0.07	-0.69	0.82	0.84	-0.52	-1.52	0.48	0.30
Traditional labor partners	0.49	-0.13	1.10	0.12	0.43	-0.62	1.48	0.41
Additional variables								
Risk proneness	-0.03	-0.13	0.06	0.50	-0.10	-0.30	0.10	0.31
Agreeableness	0.02	-0.07	0.12	0.63	-0.01	-0.20	0.19	0.89
Extraversion	-0.07	-0.18	0.05	0.24	0.10	-0.14	0.33	0.39
Sex: Male	-0.32	-1.01	0.37	0.35	0.15	-1.09	1.43	0.81
Age^{+}	-0.01	-0.03	0.02	0.58	-0.02	-0.07	0.03	0.38
Married	-0.03	-0.90	0.83	0.95	---	---	---	---
Years schooling	0.02	-0.08	0.12	0.70	-0.05	-0.18	0.08	0.45

Times to church/mo.	0.00	-0.17	0.16	0.97	0.28	-0.02	0.60	0.08

Categorical variables excluded when number of individuals at each level was too small in a given population. Survey version is excluded from the anonymous model. Being able to borrow from past communities is binned into two categories – no communities and one community in one bin, two communities in the other – for the anonymous subset. Non-anonymous sample size=101, effective sample size=27,000, DIC=368.21. Anonymous sample size=49, effective sample size=27,000; DIC=194.73. ◇Variables z-scored. +Age is centered at age 18.

Supplementary Table 7. Participants were asked, "What do other people who are not [of a given group] say about people who are [of a given group]?" (*¿Qué dicen otras personas que no son* [of a given group] *sobre las personas quienes son* [of a given group]*?*) Below are some examples of ítems that were coded as pertaining to a negative stereotype about cooperative potential, and some coded as pertaining to a positive stereotype about cooperative potential.

**Negative stereotypes**	**Positive stereotypes**
• "We leave them behind."<br>• "They work only to get by."<br>• "People say they are closed off, do not know possibilities for projects. They say they cannot express themselves because they do not make leaving the area or education a priority."<br>• "They criticize them for how they dress."<br>• "Some say the project is not working."<br>• "They speak in vain because the project never came."	• "People are jealous of their number of pigs."<br>• "People say they are rich because of cows, wood, and almonds."<br>• "They are working to get ahead."<br>• "They are preparing for a better life, reading and writing."<br>• "They say [we should] help them."<br>• "They respect them."

Supplementary Table 8. Adjustments for outliers and heteroscedasticity.

Variable	Adjustment for Statistical Purposes
Number of children living in the home	Values above 97.5 %ile rounded to 97.5 %ile
Net household income	
Places visited	
Value of market items owned	
Hours of TV or movies watched in last week	
Years of school	
Risk proneness	
Times attended church in last month	
Food insecurity	
Market items, z-scored	
Subjective SES	Logged to reduce heteroscedasticity

Supplementary Figure 1. Participants sorted cards representing local ethnic groups, churches, and work cooperatives on a scale from “groups I belong to most” to “groups I belong to least”; the yellow scale was oriented in front of the participant such that the rectangle for “groups I belong to most” was closest to him or her and the rectangle for “groups I belong to least” was farthest away. Participants had to sort all the cards on the scale, but could leave as many or as few in each rectangle as they wished. The figure below shows sorting in stages as the participant places the cards in the rectangles. The number of groups in the card sort was determined by the number of locally salient groups: 9 for the Tsimane’, 10 for the Mosetén, and 12 for the Interculturales. Some are religious organizations (e.g., Catholics are a cross, Nazarenes are a Bible), some are ethnic groups (e.g., Tsimane’ are a T, Quechua are a Q), and some are work cooperatives (e.g., the dairy cooperative has a cow, the pig farming cooperative has a pig). We classified groups placed in the two rectangles closest to the participant as in-group, those placed in the two rectangles farthest from the participant as out-group, and those in the middle rectangle as intermediate. Participant comprehension was ascertained before data were collected. Cow and pig images courtesy of johnny_automatic and tuxwrench (https://openclipart.org/detail/388/cow; https://openclipart.org/detail/216216/piggy).

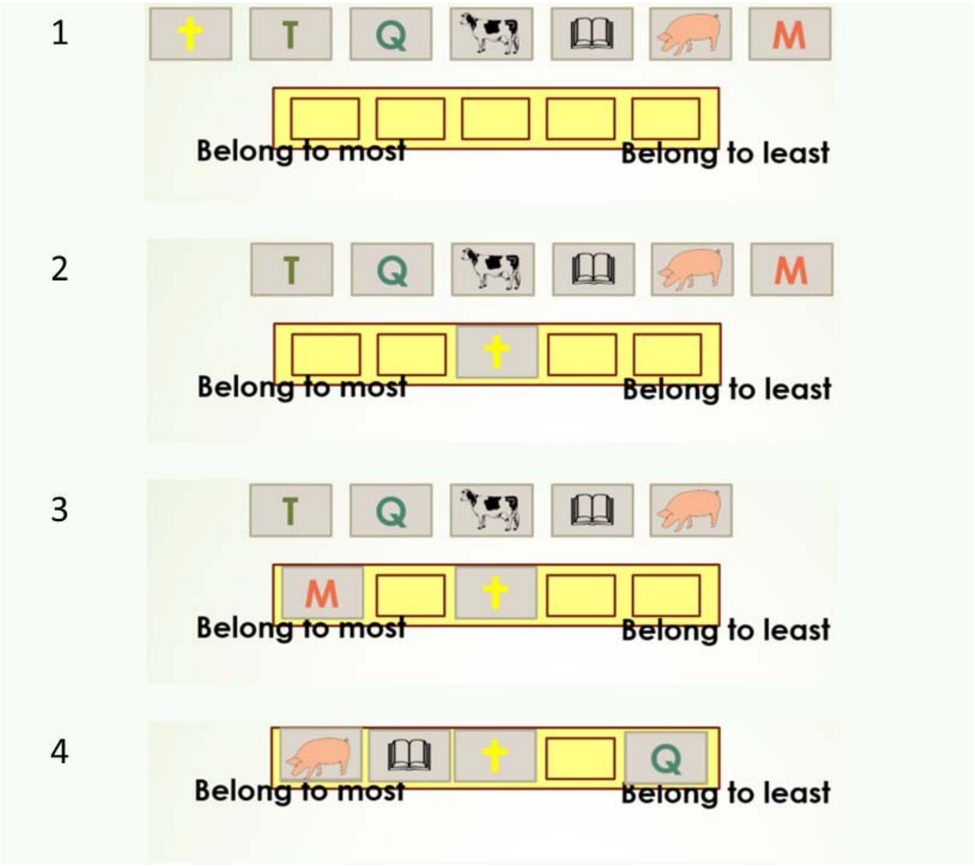
1
T
Q
M
Belong to most
Belong to least
2
T
Q
M
Belong to most
Belong to least
3
T
Q
M
Belong to most
Belong to least
4
Q
Belong to most
Belong to least

References

1. Sakel, J. Mosetén and Chimane Argument Coding: A Layered System 1. *Int. J. Am. Linguist.* **77,** 537–557 (2011).
2. Huanca, T. *Tsimane' Oral Tradition, Landscape, and Identity in Tropical Forest*. (SEPHIS – South-South Exchange Programme for Research on the History of Development, 2006).
3. Zeidler, J. A. in *Archaeology in the Lowland American Tropics* (ed. Stahl, W.) 7–41 (Cambridge University Press, 1995).
4. Corella, A., Bert, F., Pérez-Pérez, A., Gené, M. & Turbón, D. Mitochondrial DNA diversity of the Amerindian populations living in the Andean Piedmont of Bolivia: Chimane, Moseten, Aymara and Quechua. *Ann. Hum. Biol.* **34,** 34–55 (2007).
5. Bickel, G., Nord, M., Price, C., Hamilton, W. & Cook, J. Guide to Measuring Household Food Security. *Office of Analysis, Nutrition, and Evaluation* 1–82 (2000). at <naldc.nal.usda.gov/download/38369/PDF>
6. Cook, K. S. *et al.* Trust Building via Risk Taking: A Cross-Societal Experiment. *Soc. Psychol. Q.* **68,** 121–142 (2005).
7. Boyer, P. Evolutionary economics of mental time travel? *Trends Cogn. Sci.* **12,** 219–224 (2008).
8. Zaleskiewicz, T. Beyond risk seeking and risk aversion: Personality and the dual nature of economic risk taking. *Eur. J. Pers.* **15,** S105–S122 (2001).
9. Ashton, M. C. & Lee, K. Empirical, theoretical, and practical advantages of the HEXACO model of personality structure. *Personal. Soc. Psychol. Rev.* **11,** 150–66 (2007).
10. Nettle, D. The evolution of personality variation in humans and other animals. *Am. Psychol.* **61,** 622–631 (2006).
11. Sosis, R. & Ruffle, B. J. Relgious ritual and cooperation: Testing for a relationship on Israeli religious and secular kibbutzim. *Curr. Anthropol.* **44,** 713–722 (2003).
12. Shariff, A. F. & Norenzayan, A. God Is Watching You. *Psychol. Sci.* **18,** 803–809 (2007).
13. Balliet, D., Li, N. P., Macfarlan, S. J. & Van Vugt, M. Sex differences in cooperation: A meta-analytic review of social dilemmas. *Psychol. Bull.* **137,** 881–909 (2011).
14. Putnam, R. D. Bowling Alone: America's Declining Social Capital. *J. Democr.* **6,** 65–78 (1995).
15. Denissen, J. J. a & Penke, L. Motivational individual reaction norms underlying the Five-Factor model of personality: First steps towards a theory-based conceptual framework. *J. Res. Pers.* **42,** 1285–1302 (2008).
